# Association of Vitamin D Status With Mortality and Microbial Spectrum in Late-Onset Neonatal Sepsis: A Comparative Observational Study

**DOI:** 10.7759/cureus.101375

**Published:** 2026-01-12

**Authors:** Anju Yadav, Pratima Anand, Rani Gera, Leelawati Dawson

**Affiliations:** 1 Paediatrics, Employees' State Insurance Corporation (ESIC) Medical College and Hospital, Faridabad, IND; 2 Paediatrics, Lady Hardinge Medical College, New Delhi, IND; 3 Paediatrics, Vardhman Mahavir Medical College and Safdarjung Hospital, New Delhi, IND; 4 Pathology, Vardhman Mahavir Medical College and Safdarjung Hospital, New Delhi, IND

**Keywords:** culture-proven neonatal sepsis, late-onset neonatal sepsis, mortality, organism profile, vitamin d level

## Abstract

Objective

Late-onset neonatal sepsis (LONS) remains a significant cause of neonatal morbidity and mortality, especially in developing countries. Our objective was to identify vitamin D deficiency in LONS, its association with maternal vitamin D deficiency, and its relationship with microbial profile and mortality in late-onset sepsis (LOS).

Study design

We conducted an analytical observational study with two matched groups (Group A and Group B) over 29 months, from November 2018 to March 2021, in the level III outborn neonatal unit at a tertiary-care centre in New Delhi, India. A total of 320 neonates were enrolled (160 septic, 160 non-septic). Outborn neonates aged 3 to 28 days were screened; those with LOS confirmed by sepsis screen or culture were enrolled as cases, while age-matched non-septic neonates were selected as controls. Epidemiological profiles, vitamin D status, and clinical outcomes, including sepsis severity and mortality, were compared between groups.

Results

Neonatal 25(OH) vitamin D levels in Group A (20.95±18.37 ng/mL) were significantly lower than those in Group B (25.09±16.21 ng/mL) (p < 0.001). Mothers of septic neonates had significantly lower 25(OH) vitamin D levels (25.0±16.21 ng/mL) than mothers of the non-septic group (29.86±14.13 ng/mL) (p = 0.001). Vitamin D deficiency was significantly more common in the sepsis group (40.6%) compared to the non-septic group (20.6%) (p < 0.001). Gram-negative and fungal sepsis (*Acinetobacter*, *E. coli*, *Klebsiella*, and *Candida* spp.) were associated with severe vitamin D deficiency. Mortality was 23.8% in the sepsis group, with significantly lower mean vitamin D levels among non-survivors (13.9±11.9 ng/mL vs. 23.3±19.6 ng/mL, p < 0.001). Receiver operating characteristic (ROC) analysis identified a 25(OH)D cutoff of 20.85 ng/mL (AUC 0.64) for predicting LOS.

Conclusions

Neonatal and maternal vitamin D deficiency is associated with an increased risk of LONS, particularly due to gram-negative and fungal pathogens, and correlates with higher mortality.

## Introduction

Neonatal sepsis is an important cause of morbidity and mortality in newborns, especially in low- and middle-income countries. Globally, there are 6.31 million incident cases of neonatal sepsis and 0.23 million deaths due to neonatal sepsis [1]. It is characterized by signs and symptoms of infection with or without bacteraemia in the first month of life. Neonatal sepsis can be classified into early-onset sepsis (EOS) (symptoms in <72 h of age) and late-onset sepsis (LOS) (symptoms >72 h of age). Male sex, low socioeconomic status, low Apgar scores, prematurity, prolonged hospital stay, central lines, and invasive ventilation are risk factors for developing LOS [2]. The major contributing factor to increased neonatal susceptibility to infection is the immature immune system. The functional immaturity of macrophages, neutrophils, and T-lymphocytes limits their ability to mount an effective immune response [3].

Vitamin D is a fat-soluble vitamin that plays an important role in calcium metabolism, maintenance of normal calcium homeostasis, and skeletal mineralization. Recent studies have suggested vitamins as immunomodulatory and antimicrobial agents [4]. Vitamin D has an active role in the optimal functioning of the innate immune system by inducing antimicrobial peptides in epithelial cells, neutrophils, and macrophages [5,6].

Vitamin D deficiency has been linked to an increased risk of sepsis and mortality in both children and adults [7-10]. Several studies have demonstrated an association between low maternal and neonatal vitamin D levels and early-onset neonatal sepsis (EOS) [11-13]. Vitamin D deficiency has also been associated with respiratory tract infections, RSV bronchiolitis, recurrent wheezing, allergies, necrotizing enterocolitis, and EOS in neonates [14]. However, limited data exist regarding its association with LOS. This study aims to evaluate the relationship between maternal and neonatal vitamin D levels and the risk of developing late-onset neonatal sepsis (LONS). Additionally, it seeks to assess the association between vitamin D deficiency and the microbial profile of LOS, as well as its correlation with neonatal mortality.

Rationale of this study

LONS is a major cause of morbidity and mortality in newborns, especially in low- and middle-income countries like India. Despite advances in neonatal care, outcomes remain poor, and identifying modifiable risk factors is essential. Vitamin D, apart from its role in bone metabolism, plays a key role in immune regulation and antimicrobial defence. While deficiency has been linked to EOS and other infections, limited data exist on its association with LONS, microbial spectrum, and mortality. Given the high prevalence of hypovitaminosis D in Indian neonates and mothers, this study seeks to fill this gap and generate evidence that may guide preventive and therapeutic strategies.

## Materials and methods

To evaluate the association between neonatal and maternal vitamin D status and LONS, including its relationship with microbial profile and mortality.

Objectives

The primary objective was to determine the association between neonatal vitamin D deficiency and LONS. The secondary objectives were to compare maternal vitamin D levels in mothers of neonates with and without LONS, to assess the relationship between neonatal vitamin D levels and the microbial profile of LONS, and to evaluate the association between neonatal vitamin D status and mortality in LONS.

Study design and setting

This was an analytical observational study with two matched groups (Group A and Group B), conducted over 29 months, from November 2018 to March 2021, in the level III outborn neonatal unit of the Department of Pediatrics at Vardhman Mahavir Medical College and Safdarjung Hospital, New Delhi, India. The hospital is a tertiary-care academic institution with over 3,000 inpatient beds and an annual pediatric admission rate of approximately 120,000 children. The study was approved by the Institutional Ethics Committee (IEC/VMMC/SJH/Thesis/2018-10/05 dated November 6, 2018). Written informed consent was obtained from the parents or legal guardians of all enrolled neonates before participation in the study, in accordance with the Declaration of Helsinki.

Study population and study period

The study population included outborn neonates aged 3 to 28 days admitted to the level III outborn neonatal unit during the study period, from November 2018 to March 2021.

Inclusion criteria

Outborn neonates aged 3 to 28 days admitted to the level III outborn neonatal unit during the study period were eligible. Group A included neonates with a positive sepsis screen indicative of LONS (onset >72 hours of age), with or without culture positivity. Group B included age- and sex-matched neonates admitted for non-infectious conditions, with no clinical or laboratory evidence of sepsis. Written informed consent was obtained from the parents or legal guardians.

Exclusion criteria

Neonates were excluded if they had major congenital malformations or genetic syndromes, birth asphyxia (Apgar score <4 at 5 minutes or requiring prolonged resuscitation), or incomplete clinical or laboratory data relevant to the study.

Sample size 

A total sample size of 320 was included, with 160 in Group A and 160 in Group B.

For two independent, equal-sized groups, the common large-sample formula for the sample size per group is:

Where:

 Δ = μ₂ - μ₁ is the expected difference in means,

 σ is the common (pooled) SD estimate, and

 Z(1-α/2) = 1.96 for α = 0.05 (two-sided), and Z(1-β) = 0.842 for 80% power.

(Equivalently derived from the two-sample t-test/normal approximation.)

The sample size was calculated using vitamin D levels in the sepsis and non-sepsis groups reported in a previous study. In that study, the sepsis group had significantly lower mean ± SD serum vitamin D levels (13.99 ± 6.07 ng/mL) than the control group (20.56 ± 5.93 ng/mL) (p = 0.001) [15]. The difference in means was:

Δ = 20.56 - 13.99 = 6.57 ng/mL.

The pooled SD (using σ = √((s1² + s2²)/2) for planning when groups are expected to be of equal size) was calculated as follows:

s1 = 6.07, s2 = 5.93

σ ≈ √(((6.07)² + (5.93)²)/2) = 6.0004 (≈ 6.00).

Plugging into the formula:

N = (2 × (1.96 + 0.842)² × (6.0004)²) / (6.57)² = 13.10.

Rounding up, 14 subjects per group (total n = 28) would be required to detect the observed difference (6.57 ng/mL) with 80% power at α = 0.05. However, to increase the power of the study and ensure an adequate flow of patients, we included 160 participants in each group.

Methodology

This was a prospective analytical observational cross-sectional comparative study conducted in the Level III outborn neonatal unit of the Department of Paediatrics, Vardhman Mahavir Medical College and Safdarjung Hospital, New Delhi, between November 2018 and March 2021. A total of 320 outborn neonates aged 3 to 28 days were enrolled after meeting eligibility criteria. Neonates with a positive sepsis screen indicative of LOS were enrolled as Group A (n = 160) at the time of admission, concurrently with sepsis evaluation, to avoid delays and minimize variability. Vitamin D (25-OH D) levels were measured at admission, while non-septic neonates admitted for non-infectious conditions were enrolled as Group B (n = 160). Both groups were matched for postnatal age (day of life) using a ±2-day cutoff to ensure comparability.

Data were collected prospectively using a structured proforma that included prenatal, perinatal, and postnatal details. Neonatal variables recorded were age at admission, sex, gestational age, birth weight and admission weight, length, head circumference, season of birth, feeding pattern, and duration of hospital stay. Maternal demographic and clinical details were also documented. Clinical features and birth history were obtained from hospital records and verified through parental interviews.

For defining sepsis, a positive sepsis screen required the presence of two or more abnormal markers, namely total leukocyte count (TLC), absolute neutrophil count (ANC), platelet count, and CRP. Culture-proven sepsis was defined as isolation of a pathogenic organism from blood, CSF, or any other normally sterile body fluid. All definitions adhered to the National Neonatology Forum (NNF) guidelines.

Blood samples were collected once at enrolment (after sepsis diagnosis in cases). Two millilitres of venous blood were drawn aseptically from each neonate for vitamin D estimation and laboratory analysis. Serum 25-hydroxyvitamin D (25-OH D) levels were measured using an enzyme immunoassay kit (Calbiotech, USA) and reported in ng/mL. Vitamin D status was classified according to the U.S. Endocrine Society guidelines: deficiency (<10 ng/mL), insufficiency (11-30 ng/mL), and sufficiency (>30 ng/mL). Additional investigations included CBC using a Coulter-based automated hematology analyser and CRP levels estimated by the latex agglutination method in the hospital’s biochemistry laboratory. Maternal blood samples (fasting samples) for vitamin D estimation were collected within 72 hours postpartum.

Blood cultures were processed with the BD BACTEC FX40 automated system, and CSF examination was performed when there was strong clinical suspicion of meningitis or, in selected cases, with positive sepsis screens at the physician’s discretion.

Statistical analysis

Continuous variables, such as vitamin D levels, were first assessed for normality using histograms and the Kolmogorov-Smirnov test. Skewed variables were analyzed with non-parametric methods, specifically the Mann-Whitney U test, while normally distributed variables were compared using parametric tests. Categorical variables, including the primary outcome of vitamin D deficiency, were compared using the Chi-square test or Fisher’s exact test, as appropriate, and OR with 95% CI were calculated. Accordingly, multivariate logistic regression was performed to account for differences in gestation, admission weight, and maternal literacy, in addition to other clinically relevant factors, thereby minimizing confounding effects on the association between vitamin D deficiency and LOS. Receiver operating characteristic (ROC) curve analysis was conducted to assess the predictive performance of vitamin D levels for LOS. A p-value of less than 0.05 was considered statistically significant.

## Results

In the present study, various epidemiological characteristics were compared between the septic and non-septic groups (Table 1). The mean postnatal age was comparable between the sepsis and control groups. A total of 108 neonates were preterm, of whom a higher proportion were in the sepsis group (38.1%) compared to controls (29.4%). The mean birth weight was significantly lower in the sepsis group than in the control group. Male gender was more common among sepsis cases. Breastfeeding was less frequent in the sepsis group (57.5%) than in controls (68.1%), with mixed feeding more prevalent among cases.

**Table 1:** Association between blood culture findings and study groups. BLOOD C/S: Blood culture and sensitivity; CONS: Coagulase-negative staphylococci; MRSA: Methicillin-resistant Staphylococcus aureus.

BLOOD C/S	Mean	N	Std. Deviation	Minimum	Maximum
Acinetobacter	14.0375	8	11.74685	4.2	36.7
Candida	7.9167	6	1.1583	6.3	9.4
CONS	20.34	10	14.84432	6.7	46.5
E. coli	11.9579	19	8.87182	6.3	36.7
Gram-negative bacilli	8.8	2	1.13137	8	9.6
Klebsiella pneumoniae	15.7591	22	15.46368	5.6	70.2
MRSA	14.5556	9	5.62097	4.9	19.7
Staphylococcus aureus	24.5833	6	24.87701	5.3	67.3
Staphylococcus epidermidis	9.4	1	-	9.4	9.4
Sterile	27.5411	230	16.44085	4.8	114.3
Streptococcus pneumoniae	12.9857	7	4.43224	7.4	19.3
Total	23.9623	320	16.48612	4.2	114.3

The mean vitamin D level in winter was significantly lower in both the case and control groups compared to the summer and spring seasons. Neonates born in winter were significantly more likely to develop vitamin D deficiency. The mean duration of hospital stay was significantly longer among septic neonates. No significant differences were observed in mode of delivery, maternal age, or perinatal comorbidity. However, maternal education showed a significant association, with a higher percentage of mothers in the sepsis group having lower educational status.

Table 2 shows that neonates with sterile blood cultures (n = 230) had the highest mean 25-OH vitamin D level (27.54 ng/mL) and drove the overall sample mean (total mean 23.96 ng/mL, N = 320). By contrast, several pathogen groups, *Candida* (mean 7.92 ng/mL, n = 6), Gram-negative bacilli (mean 8.80 ng/mL, n = 2), and *Staphylococcus epidermidis* (mean 9.40 ng/mL, n = 1), had much lower mean vitamin D levels, while *Staphylococcus aureus* (mean 24.58 ng/mL, n = 6) and CONS (likely coagulase-negative staphylococci; mean 20.34 ng/mL, n = 10) lay between these extremes. Many groups showed large variability (e.g., *Klebsiella* mean 15.76 ng/mL, SD 15.46; sterile SD 16.44; *S. aureus* SD 24.88), and several pathogen strata had very small sample sizes, so their means are unstable. In short, sterile cultures were associated with higher mean neonatal vitamin D levels in this dataset and some infecting organisms appeared linked with lower means, but because of small sample sizes and wide SDs, formal statistical testing (and adjustment for confounders) is required before concluding any true association.

**Table 2:** Association between neonatal CRP and neonatal serum vitamin D levels. Chi-square value: 18.32; p-value: <0.001 (significant).

Neonatal serum vitamin D level (ng/mL)	≤10	10-30	30-100	Total
CRP Positive	57	50	34	141
CRP Negative	38	63	78	179
Total	95	113	112	320

The analysis in Table 3 shows a significant association between neonatal serum vitamin D levels and CRP status (χ² = 18.32, p < 0.001). Among neonates with low vitamin D (≤10 ng/mL), a higher proportion were CRP positive (57/95, 60%), indicating active infection/inflammation. In contrast, CRP positivity decreased with higher vitamin D levels, being 44% (50/113) in the 10-30 ng/mL group and only 30% (34/112) in the 30-100 ng/mL group. This suggests that lower vitamin D levels are strongly associated with a higher likelihood of CRP positivity, supporting a potential protective role of adequate vitamin D in reducing neonatal inflammatory response.

**Table 3:** Comparison of neonatal serum vitamin D levels between the two study groups. t-value: -4.67; p-value: <0.0001 (significant).

Group	Mean	N	SD	Minimum	Maximum
Sepsis	21.3959	160	18.7192	4.2	114.3
Non-sepsis	26.5287	160	13.47699	4.8	72.8
Total	23.9623	320	16.48612	4.2	114.3

The comparison of neonatal serum vitamin D levels between the study groups shows that mean vitamin D was significantly lower in neonates with sepsis (21.40 ± 18.72 ng/mL) compared to those without sepsis (26.53 ± 13.48 ng/mL) (Table 4). The difference was statistically significant (t = -4.67, p < 0.0001), indicating that vitamin D deficiency or insufficiency is more common among septic neonates, thereby suggesting a possible association between lower vitamin D levels and increased susceptibility to neonatal sepsis.

**Table 4:** Maternal serum vitamin D levels in both study groups. p-value: <0.01 (significant).

Group	Mean	N	SD	Minimum	Maximum
Case	25.514	157	16.3777	6.5	89
Control	29.986	159	14.0727	6.1	78.4
Total	27.764	316	15.4009	6.1	89

**Figure 1:**
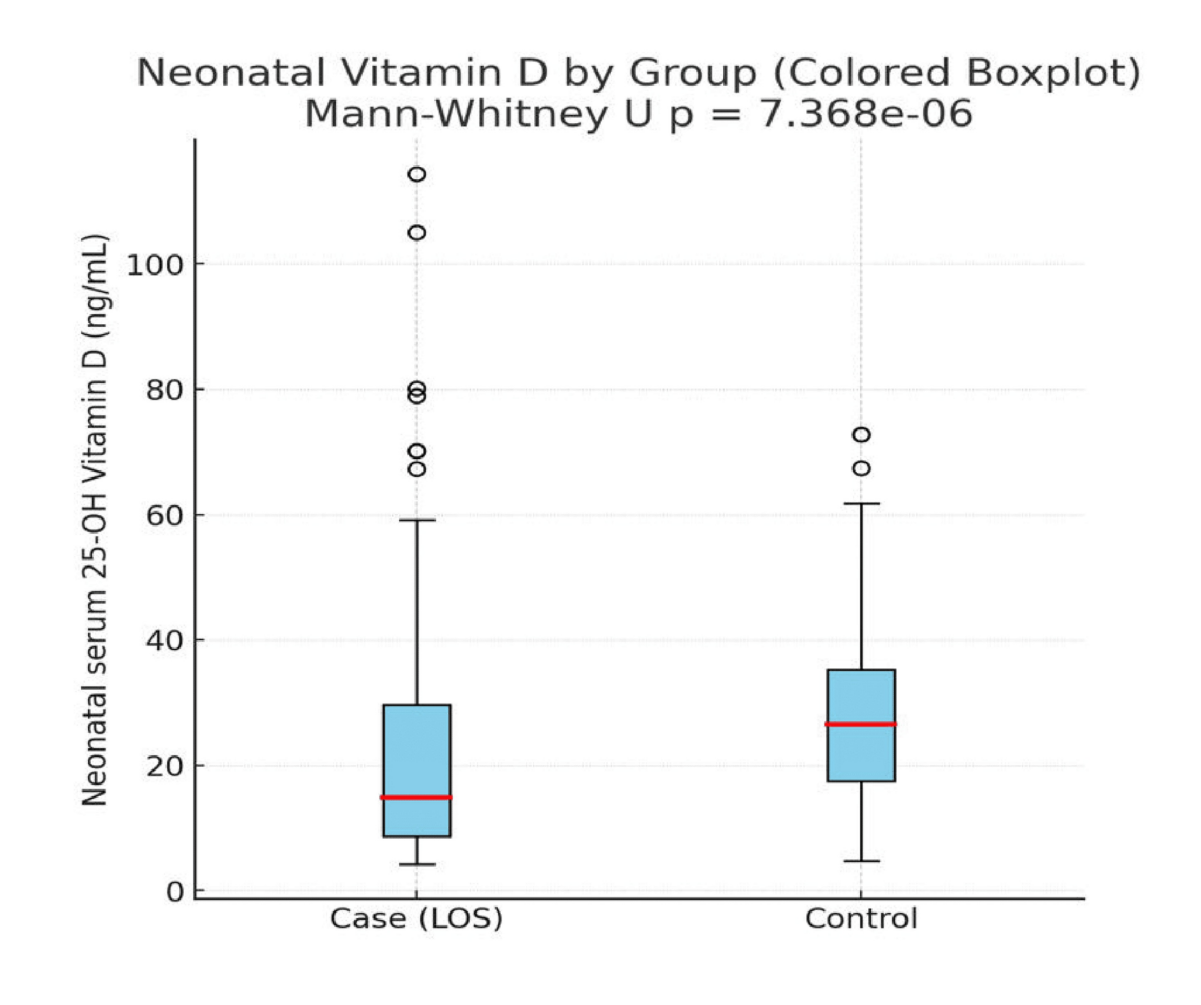
Neonatal vitamin D levels in both groups.

Figure 1 illustrates that neonates in the sepsis group had lower mean serum vitamin D levels (21.4 ng/mL) compared to the non-sepsis group (26.5 ng/mL). The error bars show wider variability in the sepsis group, reflecting greater fluctuation in vitamin D status. Overall, the graph highlights that vitamin D levels are reduced in septic neonates, supporting the significant difference observed in the statistical analysis.

Table 5 shows that mothers of neonates in the sepsis group (cases) had significantly lower mean serum vitamin D levels (25.5 ± 16.4 ng/mL) compared to mothers in the non-sepsis group (controls, 30.0 ± 14.1 ng/mL). The difference was statistically significant (p < 0.01), suggesting that maternal vitamin D deficiency may be an important contributing factor to lower neonatal vitamin D status and a higher risk of neonatal sepsis.

**Table 5:** Distribution of outcomes among study subjects in both study groups. LAMA: Left Against Medical Advice.

Outcome	Case	Control	Total
Discharge	122	149	271
Death	36	0	36
LAMA	2	11	13
Total	160	160	320

In the present study, out of a total of 320 subjects, 271 (84.7%) were discharged, 36 (11.3%) died, and 13 (4.1%) left against medical advice (LAMA) (Table 6). Among the cases, 122 (76.3%) were discharged, 36 (22.5%) died, and 2 (1.3%) left against medical advice. In contrast, all controls survived, with 149 (93.1%) discharged and 11 (6.9%) leaving against medical advice. Mortality was observed exclusively in the case group, indicating a poorer outcome compared to the control group.

**Table 6:** Epidemiologic characteristics of study participants (N = 320). LSCS: Lower Segment Caesarean Section.

Characteristics of study participants	Sepsis group (N = 160)	Control group (N = 160)	p-value
Postnatal age (Mean ± SD)	15.44 ± 7.90	15.48 ± 7.96	0.978
Gestational age category			0.098
Preterm (<37 weeks), n (%)	61 (38.1%)	47 (29.4%)	
Term (≥37 weeks), n (%)	99 (61.9%)	113 (70.6%)	
Weight (kg) (Mean ± SD)	2.46 ± 0.49	2.71 ± 0.62	0.001
Gender, n (%)			0.003
Male	99 (62.0%)	93 (58.1%)	
Female	61 (38.1%)	67 (41.9%)	
Feeding, n (%)			0.049
Breastfeeding	92 (57.5%)	109 (68.1%)	
Mixed feeding	68 (42.5%)	51 (31.9%)	
Birth season (Mean ± SD)			<0.001
Spring	30.57 ± 14.89	39.98 ± 9.08	
Summer	23.63 ± 23.16	28.08 ± 13.91	
Winter	13.57 ± 9.62	20.81 ± 10.89	
Mean duration of hospital stay (Mean ± SD)	12.55 ± 5.05	3.72 ± 1.49	<0.001
Mode of delivery, n (%)			0.718
LSCS	52 (32.5%)	49 (30.6%)	
Vaginal	108 (67.5%)	111 (69.4%)	
Maternal age (Mean ± SD)	25.39 ± 6.79	25.87 ± 6.94	0.529
Perinatal comorbidity, n (%)			0.097
None	67 (41.9%)	88 (55.0%)	
Yes	93 (58.1%)	72 (45.0%)	
Maternal education, n (%)			<0.001
Illiterate	35 (21.9%)	9 (5.6%)	
Up to 7th	47 (29.4%)	35 (21.9%)	
8th-10th	47 (29.4%)	66 (41.3%)	
>10th	31 (19.4%)	50 (31.3%)	

**Figure 2:**
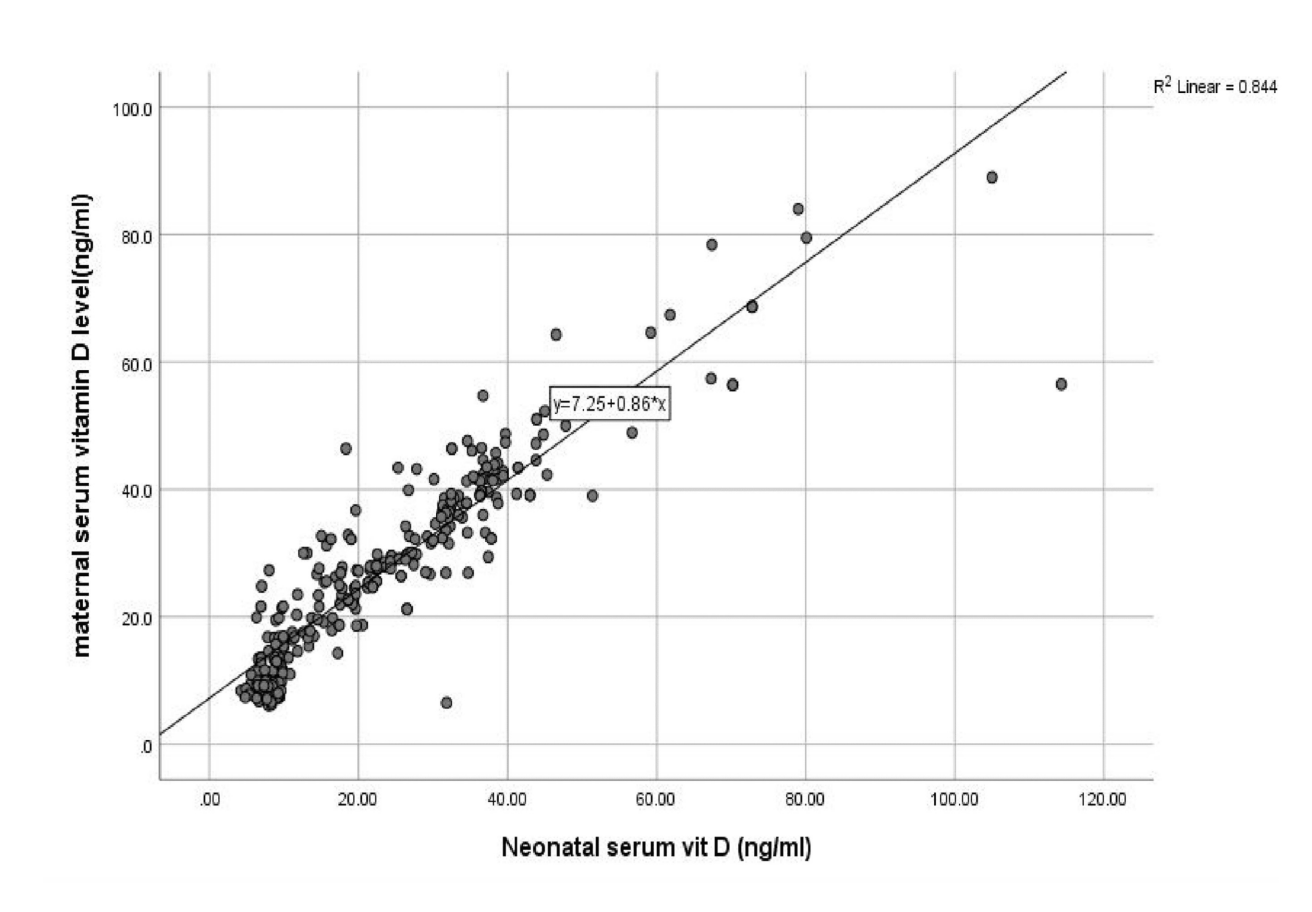
Correlation between maternal and neonatal serum vitamin D levels.

Figure 2 demonstrates a positive association between maternal and neonatal vitamin D levels, indicating that neonates born to mothers with higher serum vitamin D levels generally had higher vitamin D levels themselves. This pattern highlights the strong maternal influence on neonatal vitamin D status, suggesting that inadequate maternal vitamin D may predispose infants to deficiency and increase their vulnerability to conditions such as neonatal sepsis.

**Figure 3:**
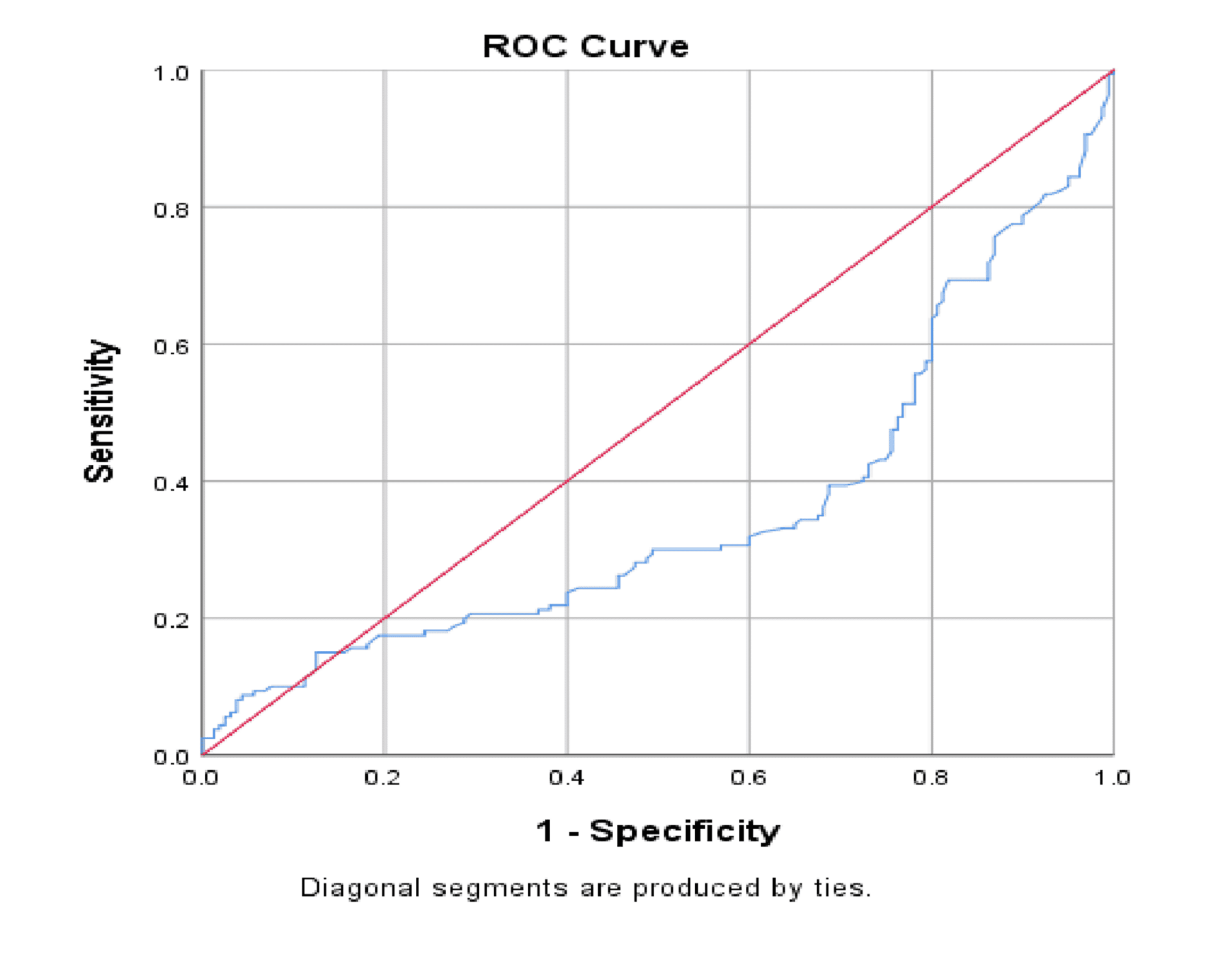
ROC curve for the cut-off of neonatal serum vitamin D levels for predicting LONS. LONS: Late-onset neonatal sepsis.

The ROC curve illustrates the diagnostic performance of neonatal serum vitamin D levels in predicting sepsis (Figure 3). The curve deviates above the diagonal reference line (line of no discrimination), indicating that vitamin D has some discriminatory ability between sepsis and non-sepsis cases. However, the curve does not reach very high sensitivity at lower false-positive rates, suggesting only moderate predictive value. The overall accuracy would be better quantified by the AUC, but visually, this ROC curve suggests that neonatal vitamin D level may serve as a supportive, though not standalone, marker for sepsis risk.

## Discussion

This analytical observational study reports vitamin D levels and their association with LOS in outborn neonates. Maternal vitamin D deficiency and lower mean vitamin D levels in neonates were found to contribute to LOS in this study. In the present study, the incidence of sepsis was higher in preterm and lower birth weight neonates compared to the non-sepsis group, consistent with Traoré FB et al. (2025) [16] in Sub-Saharan Africa and Das M et al. (2024) [17], both of whom identified prematurity and low birth weight as major risk factors for neonatal sepsis. Exclusive breastfeeding was less frequent among septic neonates in our cohort, supporting evidence from the Lancet Breastfeeding Series (2016) [18] and Raihana S et al. (2019) [19], who both demonstrated a protective effect of early and exclusive breastfeeding against severe neonatal illness.

Seasonal variation was evident, with significantly lower vitamin D levels in winter-born neonates. This matches general paediatric literature showing seasonal declines in 25(OH)D due to reduced UV exposure. Sepsis was also associated with a significantly longer hospital stay, consistent with Das M et al. (2024) [17], who showed prolonged admissions among septic neonates. By contrast, no significant association was found with maternal age, mode of delivery, or perinatal comorbidity, in line with Salama B and Tharwat EM (2023) [20], who also did not identify these factors as independent risks. Importantly, lower maternal education was significantly linked to neonatal sepsis, echoing Sturrock S et al. (2023) [21], who highlighted education as a key social determinant of neonatal outcomes.

Analysis of blood culture profiles revealed that neonates with sterile cultures had the highest mean vitamin D levels, supporting observations by Ozdemir AA and Cag Y et al. [22] that adequate vitamin D protects against invasive sepsis, while Cutuli SL et al. (2021) [23,24] summarized multiple cohorts showing that deficiency is strongly associated with culture positivity. Gram-negative infections such as *E. coli* and *Klebsiella* were also linked to lower vitamin D, consistent with Cutuli SL’s review and experimental work showing that vitamin D reduces Gram-negative virulence and biofilm formation. CONS and *S. aureus* showed intermediate to variable vitamin D levels, reflecting their prominence in LOS and complex interactions with host immunity, as reported by neonatal series and mechanistic studies. Notably, *Klebsiella* showed wide variability in mean vitamin D, mirroring epidemiologic reports of heterogeneity across Gram-negative infections. Finally, the small sample size in several pathogen subgroups limits definitive organism-specific conclusions, a limitation also noted in prior neonatal sepsis studies, highlighting the need for larger multicentre investigations.

Maternal vitamin D status also showed a significant association with neonatal outcomes. Mothers of septic neonates had lower vitamin D levels (25.5 ± 16.4 ng/mL) than those of controls (30.0 ± 14.1 ng/mL; p < 0.01). Moreover, neonatal vitamin D levels positively correlated with maternal levels, reinforcing the strong maternal influence on neonatal vitamin D status, as highlighted in earlier studies.

Analysis of inflammatory markers further strengthened the association: CRP positivity was highest among neonates with severe vitamin D deficiency (≤10 ng/mL), while those with higher vitamin D levels (30-100 ng/mL) had markedly lower CRP positivity (30%). This inverse trend suggests a protective immunomodulatory role of vitamin D against neonatal inflammation and sepsis.

The bacteriological profile revealed culture positivity in 41.9% of LONS cases. Coagulase-negative staphylococci (CONS) were most common, followed by MRSA, *E. coli*, and *Klebsiella spp.*, mirroring findings by Rashmi P and Praveen BK [25] and Singh P and Chaudhari V [26], where CONS and MRSA predominated in LOS. Vitamin D levels varied significantly across pathogens: Gram-negative organisms and fungi were associated with the lowest levels (e.g., *E. coli* 11.95 ng/mL, *Klebsiella* 15.7 ng/mL, *Candida* 7.9 ng/mL, *Acinetobacter* 14.0 ng/mL), whereas sterile cultures had the highest mean levels (27.5 ng/mL). This trend suggests that severe vitamin D deficiency may predispose to invasive infections by more virulent organisms.

Our ROC curve analysis further demonstrated moderate predictive utility of neonatal vitamin D levels in distinguishing sepsis from non-sepsis. While not a standalone marker, it may be valuable as part of a broader risk stratification tool.

The epidemiological profile in our cohort revealed a predominance of male neonates (62%) and preterm births (38.1%) among the septic group, consistent with prior studies showing male vulnerability and higher risk with prematurity. Feeding practices also suggested a protective role, with exclusive breastfeeding being less prevalent among septic neonates, aligning with the protective effect reported by Kaur A et al. [27]. Seasonal variation was evident: septic neonates born in winter had the lowest vitamin D levels (13.6 ng/mL), consistent with Singh P and Chaudhari Vs observations of seasonal fluctuations [26]. We also found an association between lower maternal education and neonatal sepsis, suggesting socioeconomic and behavioural influences on neonatal outcomes, as reported by Wang M et al. [28].

In our study of 320 subjects, mortality was observed only in the case group (36/160, i.e., 22.5%), whereas none of the controls died. This stark contrast underscores a substantially worse outcome in the case group. Published studies in obstetrics and neonatology also reflect such disparities in outcomes between high-risk and lower-risk groups.

For example, Avelino IC et al. (2024) [29] conducted a matched case-control study in a neonatal referral centre and identified key determinants of neonatal mortality. They matched each neonatal death with two surviving infants and found that factors such as prematurity, infection, low birth weight, and lack of adequate antenatal care significantly increased mortality risk in the case cohort versus controls. Another recent work by Fink DA et al. (2023) [30] examined trends in maternal mortality and severe maternal morbidity (SMM) over time in U.S. hospital deliveries. They reported a decline in in-hospital maternal mortality from 2008 to 2021, but a concurrent increase in SMM rates, suggesting that a higher burden of complications increases the risk trajectory (JAMA Network). Importantly, adverse outcomes correlated with vitamin D status: neonates who died had significantly lower vitamin D levels (13.9 ± 11.9 ng/mL) compared to survivors (23.3 ± 19.6 ng/mL). Blood culture positivity and meningitis were also more frequent in vitamin D-deficient neonates, indicating a potential role of deficiency in the severity and complications of sepsis.

Limitations

This study has certain limitations. First, its observational cross-sectional design limits causal inference, as vitamin D levels were measured after the onset of sepsis, raising the possibility of reverse causation. Second, being a single-centre study conducted in an outborn tertiary unit, the findings may have limited generalisability to inborn or community neonatal populations. Third, although the overall sample size was adequate, small numbers within individual pathogen subgroups, particularly Gram-negative and fungal infections, limit the robustness of organism-specific associations. Fourth, several potential confounders influencing vitamin D status, such as maternal supplementation, dietary intake, sunlight exposure, and vitamin D-binding protein levels, were not assessed, and residual confounding cannot be excluded despite multivariate adjustment. Finally, the moderate predictive performance of vitamin D levels on ROC analysis indicates that vitamin D should be viewed as a supportive risk marker rather than a standalone diagnostic or prognostic tool.

## Conclusions

This study demonstrates that vitamin D deficiency is significantly associated with late-onset neonatal sepsis, its severity, and adverse outcomes, including mortality and meningitis. Lower levels were especially pronounced in culture-positive cases and in infections with virulent organisms such as Gram-negative bacilli and fungi. These findings highlight the need for larger multicentre studies and interventional trials to evaluate whether maternal and neonatal vitamin D supplementation could serve as a preventive strategy against neonatal sepsis. Screening for and early correction of hypovitaminosis D, particularly in preterm and high-risk neonates, may reduce infection-related morbidity and mortality.
